# Coalescent Method in Conjunction with Niche Modeling Reveals Cryptic Diversity among Centipedes in the Western Ghats of South India

**DOI:** 10.1371/journal.pone.0042225

**Published:** 2012-08-02

**Authors:** Jahnavi Joshi, K. Praveen Karanth

**Affiliations:** 1 Centre for Ecological Sciences, Indian Institute of Science, Bangalore, Karnataka, India; Institut de Biologia Evolutiva - Universitat Pompeu Fabra, Spain

## Abstract

**Background:**

There has been growing interest in integrative taxonomy that uses data from multiple disciplines for species delimitation. Typically, in such studies, monophyly is taken as a proxy for taxonomic distinctiveness and these units are treated as potential species. However, monophyly could arise due to stochastic processes. Thus here, we have employed a recently developed tool based on coalescent approach to ascertain the taxonomic distinctiveness of various monophyletic units. Subsequently, the species status of these taxonomic units was further tested using corroborative evidence from morphology and ecology. This inter-disciplinary approach was implemented on endemic centipedes of the genus Digitipes (Attems 1930) from the Western Ghats (WG) biodiversity hotspot of India. The species of the genus Digitipes are morphologically conserved, despite their ancient late Cretaceous origin.

**Principal Findings:**

Our coalescent analysis based on mitochondrial dataset indicated the presence of nine putative species. The integrative approach, which includes nuclear, morphology, and climate datasets supported distinctiveness of eight putative species, of which three represent described species and five were new species. Among the five new species, three were morphologically cryptic species, emphasizing the effectiveness of this approach in discovering cryptic diversity in less explored areas of the tropics like the WG. In addition, species pairs showed variable divergence along the molecular, morphological and climate axes.

**Conclusions:**

A multidisciplinary approach illustrated here is successful in discovering cryptic diversity with an indication that the current estimates of invertebrate species richness for the WG might have been underestimated. Additionally, the importance of measuring multiple secondary properties of species while defining species boundaries was highlighted given variable divergence of each species pair across the disciplines.

## Introduction

Delimiting species and reconstructing their phylogenetic histories is central to systematic biology [1]. It is essential to delineate species boundaries as they are fundamental units in biogeography, ecology and evolutionary studies [2], [3]. Species are units at which micro-evolutionary processes operate and can be potentially studied [2]. Traditional morphology based taxonomy is very useful and has been widely used, only failing to distinguish species in cases of closely related taxa, very recent radiations, or in taxonomic groups that exhibit morphological stasis [4], [5]. In such cases, molecular tools provide an independent suite of characters and additional data with which one can delimit species [5], [6], [7]. In the recent years, ecological niche modelling has also been useful in species delimitation and new species discovery [8], [9], [10], [11], [12]. Thus, the contemporary approach involves integrative taxonomy that uses multiple lines of evidence to delineate species boundaries, particularly in the case of taxonomically problematic groups. This approach also provides the operational basis for de Queiroz (2005) lineage species concept which attempts to present a unified synthesis of various species concepts and recommends the use of multiple lines of evidence for species delimitation. The need for an integrated approach for species delimitation has been justified both conceptually [2], [4], [13], [14], [15] as well as empirically across taxonomic groups (e.g. arthropods [4] and references therein, birds [16], plants [17], snakes [18]).

Of the studies that have employed an integrative approach to species delimitation, and included molecular data have largely used monophyly as a proxy for taxonomic distinctiveness [9], [19], [20]. An integrated approach also uses monophyly as a basic criterion, followed by diagnosis/evaluation of its uniqueness through multiple data sources. However, monophyly or lack of it, cannot be unambiguously used to assess taxonomic distinctiveness, because these patterns can be generated by stochastic processes of lineage [21], [22]. The recent past has seen development of rigorous statistical methodologies to evaluate monophyletic units in a coalescent framework [21], [22], [23], [24]. For example, Rosenberg (2007) developed a test that calculates the probability (Rosenberg’s p) of observed monophyly being a true pattern and not a product of random branching. Nevertheless, some species might exhibit deep intraspecific divergence solely due to the stochastic process of gene coalescence. In such situations one might erroneously infer the presence of a cryptic species. Rodrigo *et al.* (2008) have proposed a statistical measure to address this issue of panmixis that tests whether the observed pattern could have arisen under a standard coalescent model (null model). With the help of the aforementioned tests, *a priori* identification of statistically supported putative species can be carried out and their species status can be further evaluated through other datasets based on morphology, behavior and ecology in an integrative framework.

The Western Ghats (WG), a chain of mountain ranges along the west coast of peninsular India (PI), provides us with an ideal setting for implementing such an integrative approach in tropical Asia. This is because the WG exhibit high levels of species richness and endemism and are one of the biodiversity hotspots of tropical Asia [25]. Secondly, many new species have been described from this region in the recent past [26], [27], [28], suggesting a possibility of many more still remaining undiscovered. More importantly, most of the new species discoveries were based only on evidence from morphological data, thus this region might be harbouring many morphologically cryptic species. While the use of molecular data to describe new species has been limited in the WG [29], niche modeling has been used in only a few studies, and only to predict species distributional limits [30], [31]. However, integration of these three datasets, i.e. morphological, molecular and ecological data, for species delimitation is lacking for the WG biota. Wiens and Penkrot (2002) posed an intriguing question that is especially relevant in the WG scenario, “Will DNA sequence phylogenies give a very different picture of species diversity and patterns of speciation from that obtained from traditional morphological characters?” The invertebrate fauna of the WG are of special interest as it has been poorly studied, has a number of taxonomically challenging groups, and even basic information on species diversity and distributions is lacking. For example, in the case of centipedes (Arthropoda: Chilopoda) there have only been a few studies in the WG. Most of the studies produced species checklists for some parts of the WG [32], [33]. Furthermore among centipedes, species identification based on external morphological characters has been problematic [34], [35]. A recent phylogenetic study also indicated the lack of morphological variation among deeply divergent clades of Scolopendrid centipedes of peninsular India [36]. Lately, anatomical characters have also been used in the Chilopodan systematics. However, the use of these characters is limited as it involves dissections and detailed descriptions of specific internal organs of the species under consideration [34]. As mentioned before, in such cases where external morphological characters are not adequate for species delimitation, use of molecular and ecological datasets might be imperative. Hence, the usefulness of an integrative approach was explored in characterizing the diversity among the centipedes of the genus *Digitipes* (Family: Scolopendridae) in the WG.

The genus *Digitipes* was initially known only from central parts of the Congo Basin in Africa where three endemic species were described [37]. Six new species of centipedes endemic to WG described in 1984 were also assigned to this genus [38]. These descriptions demonstrated low morphological variation within the species of the genus, i.e., very few diagnostic characters could be used to distinguish species. Furthermore, very little information about their distribution and habitat requirements was provided. Two recent surveys in southern parts of the WG have not contributed much to our understanding of their distribution [32], [33]. Thus, a systematic study evaluating taxonomic status of these species and their distributional ranges encompassing the entire WG is needed. In a recent molecular phylogenetic study of Indian Scolopendrid centipedes, species of the genus *Digitipes* from the WG formed a distinct clade that was sister to a clade comprising the three genera – *Rhysida, Ethmostigmus* and *Alipes* [36]. Thus, the Indian members of the genus *Digitipes* might represent an endemic radiation that could be potentially used to study underlying mechanisms of speciation and diversification in the WG. More importantly, the divergence date estimates pointed at its Gondwanan origin with the earliest estimate within Indian *Digitipes* falling in the late Cretaceous. Thus, in spite of their antiquity, Indian members of the genus *Digitipes* are morphologically less variable and have a restricted distribution pattern in the WG. These attributes, compounded by how little is understood of the distribution of the Indian species in the genus *Digitipes*, makes this group an ideal candidate for exploring the integrative approach outlined above.

To evaluate molecular divergence two mitochondrial DNA markers (mtDNA) cytochrome *c* oxidase I (COI) and 16S ribosomal gene (16S rDNA) and one nuclear 28S ribosomal gene (28S rDNA) were sequenced for *Digitipes* samples collected from across their distributional range. These sequences were subjected to phylogenetic analyses to identify well supported clades. Two different coalescent approach based methods were in-turn implemented on both the mitochondrial genes, COI and 16S rDNA for *a priori* identification of putative species. Morphological and ecological data were then used to assess the uniqueness of these putative species independently and to delineate species boundaries. Morphological variations were evaluated by measuring 11 morphometric characters. Continuous environmental (bioclimatic) variables were used to investigate differentiation along the ecological axis.

## Results

### Summary of Molecular, Morphological and Ecological Niche Analysis

#### mtDNA phylogeny

**Figure 1:**
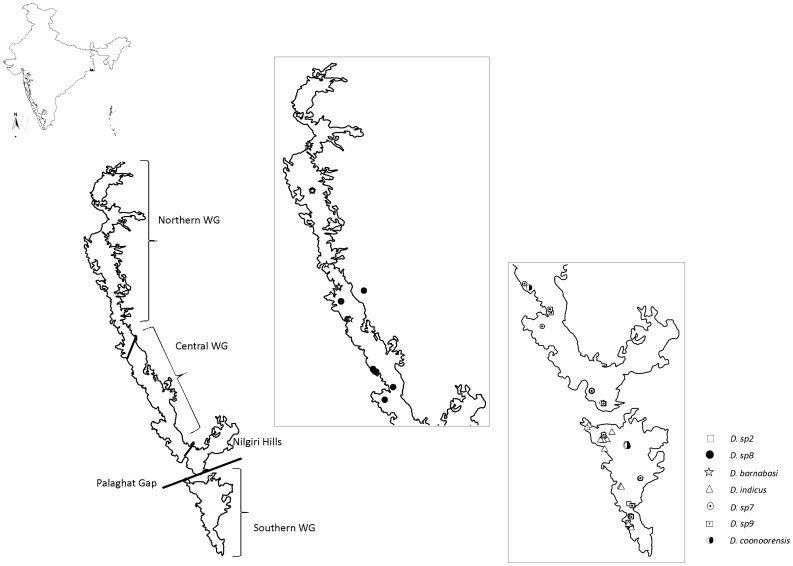
Sampling localities of the genus *Digitipes* in the Western Ghats (WG). Boundaries of northern, central and southern WG and Nilgiris are also shown.

**Figure 2:**
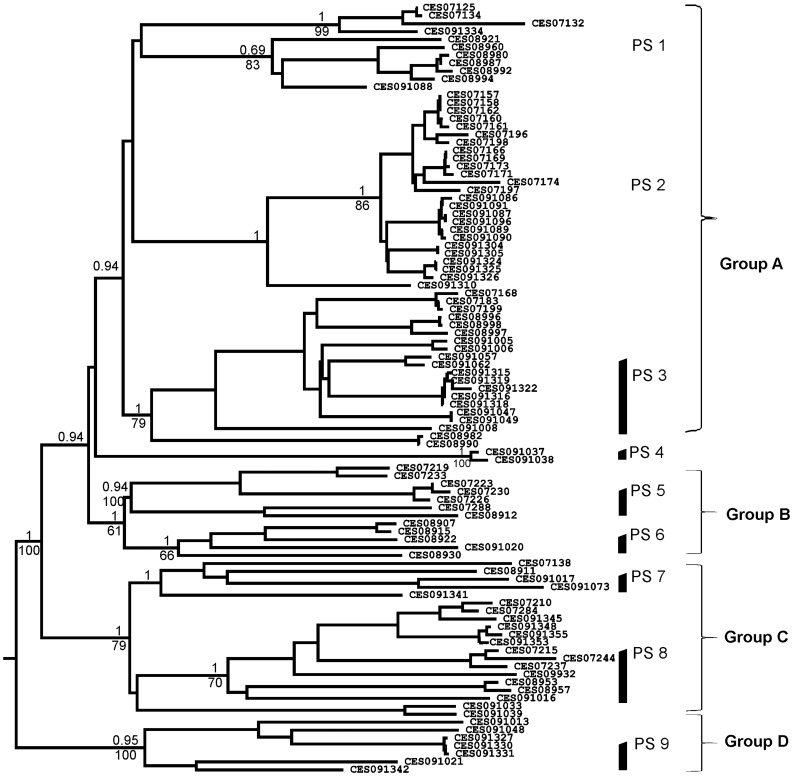
Minimum Evolution tree based on mtDNA markers with Bayesian posterior probabilities and Parsimony Bootstrap values (only >50% support values shown).

The sequences of two mitochondrial markers, COI Parsimony Informative Sites (PIS) - 213/543 and 16S rDNA (PIS - 205/455) for 97 *Digitipes* specimens from 42 localities across the WG were obtained (Fig. 1, Table S1). All three approaches, Bayesian, Parsimony and minimum evolution (ME) retrieved very similar tree topologies. Subsequently, topological congruence among these trees was assessed through Shimodaira-Hasegawa (SH) test which was not significant (p = 0.151). Therefore, only the ME tree is shown, along with the Bayesian posterior probabilities, Parsimony bootstrap support for various nodes (Fig. 2). Four major clades were retrieved in the ME tree, namely A, B, C and D. Among these four clades, clade D received highest posterior probability (PP) and bootstrap support (BS), while each of the remaining clades received high PP between 1.0 to 0.94 and low BS values (Fig. 2). Clade D was sister to the clades A, B and C received high PP and BS. Within the group containing the clades A, B and C, clade A and B were sister (PP of 0.94 and BS of less than 50%) and the clade C was sister to this group. In addition, a clade comprising two individuals belonging to putative species (PS) 4 was also recovered but its phylogenetic position was not resolved.

All major clades except D contained multiple sub-clades. These sub-clades along with clade D were subjected to coalescent test where monophyly of each of these units was assessed by using genes, COI and 16S independently (Table S2). This exploratory analysis indicated presence of nine putative species, PS1 to PS9 (Fig. 2). In all the PS the probability of obtaining monophyly by chance was not significant (Rosenberg’s p<0.05). Rodrigo’s p was close to 1 for all putative species, except PS2 in COI and PS1 and PS2 in 16S, indicating no cryptic species within any of these putative species.

Within clade A, three putative species (sub-clades) were recovered including PS1, PS2 and PS3 with PS1 and PS2 being sister to each other. The monophyly of PS2 and PS3 was well supported whereas PS1 received low support (Fig. 2). In clade B, PS5 and PS6 were reciprocally monophyletic receiving good support. Within group C, PS8 was monophyletic with 70% of BS and PP of 1, whereas there was no support for the monophyly of PS7 due to the ambiguous position of CES091033 and CES091039. In the Bayesian tree these two specimens branched with PS8 with poor support, but in the parsimony tree with PS7. Additionally in COI tree (not shown) they branch with PS7 with high support and in the nuclear tree CES091033 branched with PS7 (see below). Therefore, CES091033 and CES091039 were considered as part of PS7. Clade D corresponded to a single species, i.e. PS 9 with PP of 0.95 and 100% BS.

Only three putative species were assigned to previously described species based on traditional taxomony and specimens from type localities –*Digitipes coonoorensis* (Jangi and Dass, 1984) (PS1), *D. barnabasi* (Jangi and Dass, 1984) (PS8) and *D. indicus* (Jangi and Dass, 1984) (PS2), while the other six PS are potentially new species. Among these six species, PS4 was represented only by two individuals thus, do not discuss it further in this paper.

#### Nuclear phylogeny

**Figure 3:**
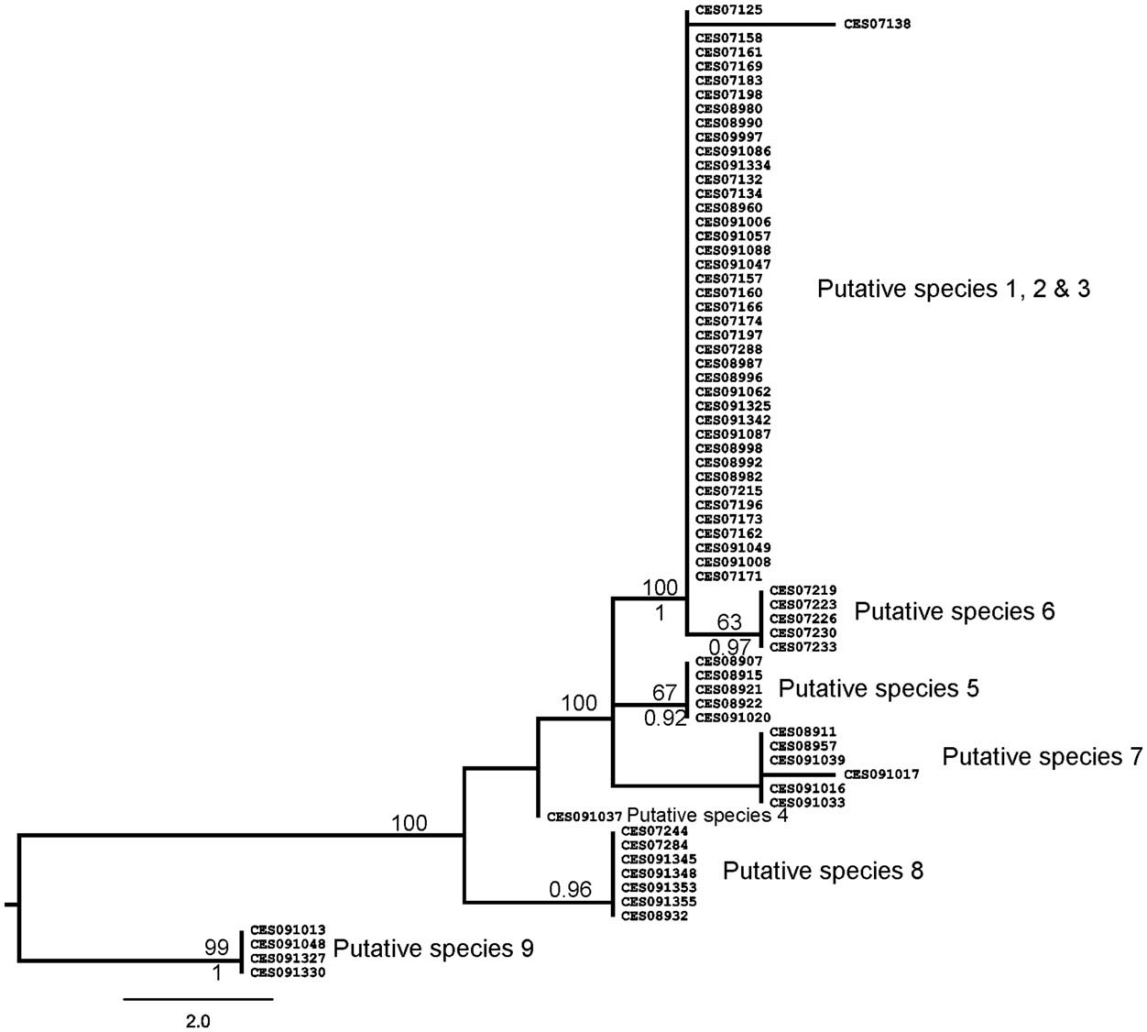
Minimum Evolution tree based on nuclear ribosomal gene with Bayesian posterior probabilities and Parsimony Bootstrap values (only >50% support values shown).

The MP, ME and Bayesian analyses retrieved largely similar tree topology, which was confirmed by SH test (p = 0.99). The ME tree is presented with parsimony bootstrap and Bayesian posterior probability. The 28S rDNA tree retrieved six out of the nine putative species of the mtDNA tree, but the relationship among these species was different (Fig. 3). Putative species PS1, PS2 & PS3 did not retrieved in the nuclear tree, nevertheless members of these three species clustered together with high PP and BS, this group corresponded to clade A of mtDNA tree. Monophyly of PS5 & PS6 received BS of 65% and 63% and PP of 0.92 and 0.97 respectively, though sister-relationship among these putative species was not recovered. PS4, PS7 and PS8 were recovered as distinct clades but with low support, whereas PS9 received high support.

#### Morphological analysis

**Figure 4:**
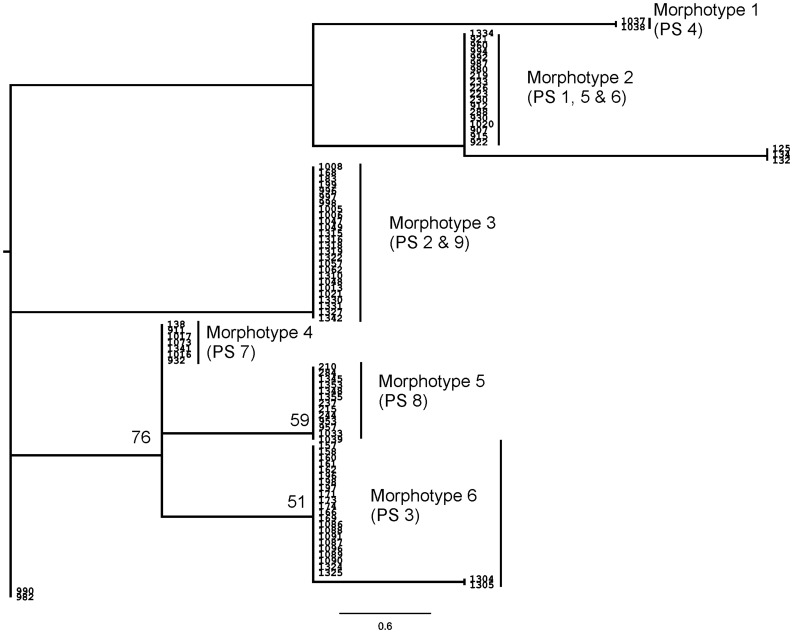
Neighbour-Joining (NJ) tree based on morphology with NJ bootstrap values (only >50% support values shown).

A total of 11 morphological characters were examined for 95 individuals (see Materials and Methods for the details). Neighbor-Joining (NJ) tree retrieved six clusters and these morphotypes were numbered from 1–6 (Fig. 4). Morphotypes 1, 4, 5 and 6 corresponded to PS4, PS7, PS8 and PS3 respectively. Morphotype 2 comprised of individuals belonging to PS1, PS5 & PS6 and morphotype 3 had individuals from PS2 and PS9. However, two individuals belonging to PS2, CES08990 and CES08982 were unresolved. In total the morphological analysis retrieved four out of the nine PS (subclades) of the mtDNA phylogeny. Comparison of specimens with original species descriptions and sampling of topotypical material allowed us to assign species names to PS1, PS3 and PS8 as *D. coonoorensis*, *D. indicus* and *D. barnabasi* respectively.

#### Ecological niche modeling and principle component analysis

**Figure 5:**
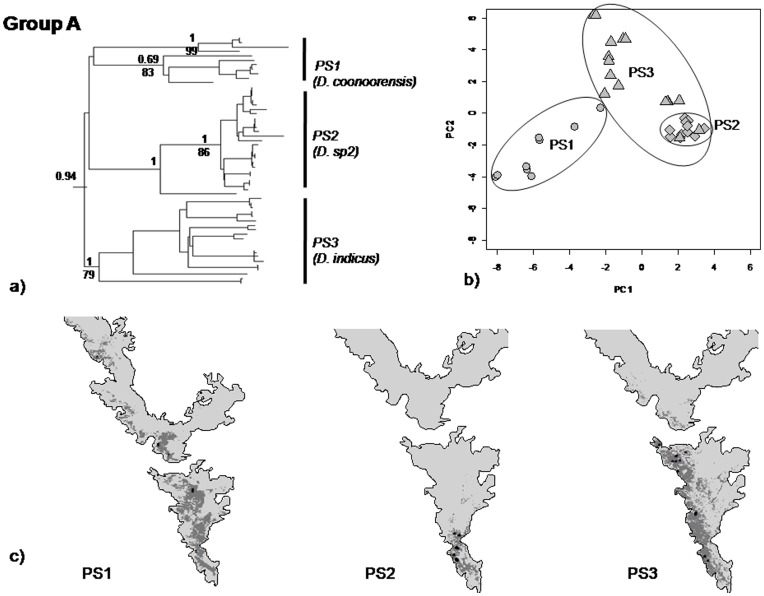
mtDNA phylogeny, principal component analysis (PCA), and predicted and current distribution maps for PS1, PS2 and PS3. (a) mtDNA phylogeny; (b) PCA - in this plot grey circles represents PS1, diamonds-PS2 and triangles-PS3 respectively; (c) Predicted and current distribution maps in the southern and central Western Ghats.

**Figure 6:**
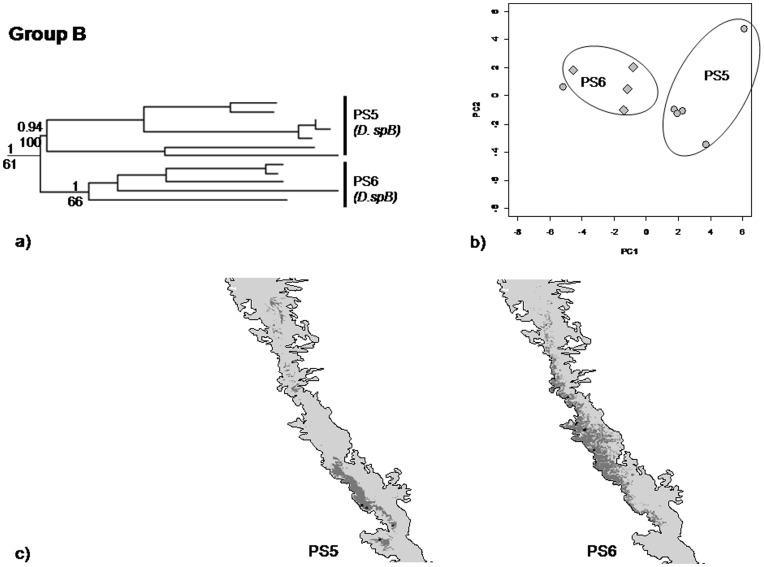
mtDNA phylogeny, principal component analysis (PCA), and predicted and current distribution map for PS5 and PS6. (a) mtDNA phylogeny; (b) PCA - in this plot grey circles represents PS 5 and diamonds PS 6 respectively; (c) Predicted and current distribution maps in the central Western Ghats.

**Figure 7:**
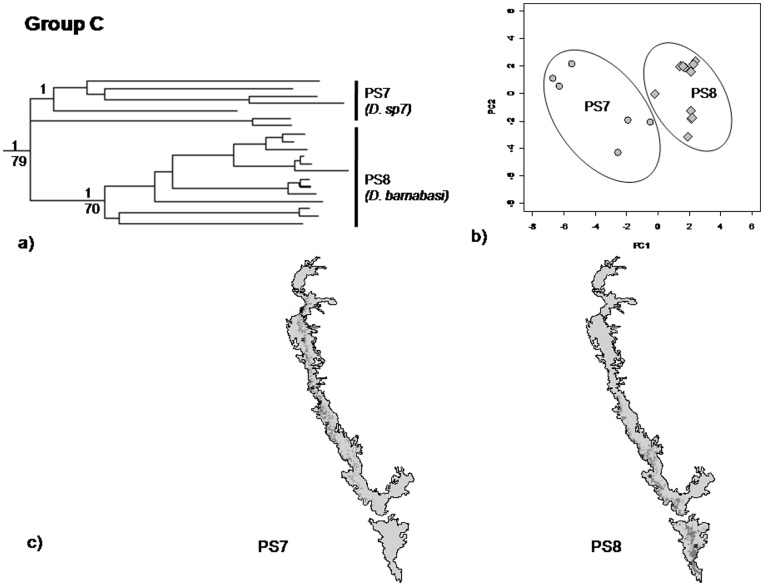
mtDNA phylogeny, principal component analysis (PCA), and predicted and current distribution maps for PS7 and PS8. (a) mtDNA phylogeny; (b) PCA - in this plot grey circles represents PS 7 and diamonds-PS 8 respectively; (c) Predicted and current distribution maps in the Western Ghats.

The area of predicted distribution varied across species, some species matched the currently known distribution, for some others the predicted range extended beyond their known distribution. Sampling localities along with predicted distribution maps for each species pair are shown (Fig. 5, 6 and 7). Overlap in the predicted distribution ranged from 19–46% across species pairs. The loadings of principle component 1 and 2 for each species pair are summarized in Table 1. The results of environmental envelop (MANOVA) and geographic overlaps (predicted distributions) are discussed below.

1. Clade A PS1, PS2 and PS3– Predicted distributions of PS2 and PS3 were similar to the current distributions with few additional areas. In case of PS1, the predicted distribution had reported more areas in the southern WG (SWG). Overlap in predicted areas between PS1 and PS2 was 19% and largely in the SWG, for PS1 and PS3 the overlap was 46%, again in the southern parts of WG. In contrast, the overlap between PS2 and PS3 was only 12%. In PCA, PC1 (51%) and PC2 (33.7%) explained 85% variation. PS1 and PS2 were well separated on PC1 and PS3 was separated from PS1 and PS2 on PC2 (Fig. 5, Table 1). There were significant differences in environmental envelopes of PS1, PS2 and PS3, for both PC1 and PC2 (MANOVA, p<0.001).
2. Clade B; PS5 and PS6– Predicted distributions of PS5 and PS6 overlapped substantially (46%) in the central parts of the WG (Fig. 6). PC1 (56.5%) and PC2 (23%) explained 89% variation in the PCA plot. In the PCA, though they responded to different sets of bioclimatic variables, the difference was not significant (MANOVA, p = 0.06).
3. Clade C; PS7 and PS8– There was 27% overlap in the predicted distributions of PS7 and PS8. Their ranges overlapped in the Central parts of WG (Fig. 7, Table 1). PC1 (50%) and PC2 (23%) explained 73% of the variation in the distribution of PS7 and PS8. On the PCA plot 73% of the variation was explained by first two axes, PS7 and PS8 were separated on PC1 and explained 50% variation and the remaining variation was explained by PC2 i.e. 23%. In the PCA, the response to the bioclimatic variables was significantly different (MANOVA, p<0.001).

**Table 1:** Summarized PCA loadings for each species pair.

Bioclim layers		Species 1, 2 and 3		Species 5 and 6		Species 7 and 8	
		PC1	PC2	PC1	PC2	PC1	PC2
Annual Mean Temperature	bio1	0.243485	0.252741	0.285926	−0.14883	0.263457	−0.22186
Mean Temperature ofWarmest Quarter	bio10	0.233272	0.268578	0.296759	−0.08402	0.297515	−0.13277
Mean Temperature ofColdest Quarter	bio11	0.244618	0.250533	0.273859	−0.19558	0.20523	−0.3407
Annual Precipitation	bio12	−0.21354	0.235532	−0.2306	−0.29294	0.009383	−0.22047
Precipitation of WettestMonth	bio13	−0.25383	0.221925	−0.21033	−0.30665	0.130338	−0.12817
Precipitation of Driest Month	bio14	0.155093	−0.2892	−0.15162	0.04335	−0.29766	−0.06042
Precipitation Seasonality	bio15	−0.25586	0.228317	−0.11018	−0.3149	0.265538	0.04854
Precipitation of WettestQuarter	bio16	−0.23999	0.234138	−0.2128	−0.31343	0.104951	−0.14725
Precipitation of Driest Quarter	bio17	0.197457	−0.23771	−0.2372	0.219353	−0.30057	−0.07925
Precipitation of WarmestQuarter	bio18	−0.17313	0.01229	−0.21057	−0.04135	−0.2031	0.050122
Precipitation of ColdestQuarter	bio19	0.004545	0.341469	−0.21752	0.062863	−0.14478	−0.27939
Mean Diurnal Range	bio2	−0.23347	0.154012	0.254126	0.130471	0.259663	0.247837
Isothermality	bio3	0.298745	−0.00222	0.098035	−0.3267	−0.23034	−0.27088
Temperature Seasonality	bio4	−0.2248	0.193612	0.087565	0.379179	0.234622	0.30479
Max Temperature ofWarmest Month	bio5	0.20508	0.296498	0.300038	0.000383	0.311578	−0.03605
Min Temperature ofColdest Month	bio6	0.257638	0.232011	0.240068	−0.24953	0.057688	−0.45441
Temperature Annual Range	bio7	−0.26704	0.116835	0.193177	0.345706	0.257762	0.284314
Mean Temperature ofWettest Quarter	bio8	0.268169	0.210283	0.281687	−0.16948	0.244431	−0.19273
Mean Temperature ofDriest Quarter	bio9	0.233189	0.268013	0.288547	−0.14696	0.240981	−0.28987

## Discussion

### Review of the Systematics of the Genus Digitipes from WG

In the following section, the evidence from multiple datasets is integrated for each species group (clades) and assessed the status of PS along within these groups.

#### (1) Clade A; PS1, PS2 and PS3

In the mtDNA tree, PS1, PS2 and PS3 were retrieved as distinct clades. Coalescent analysis also supported the same showing very low divergence within species and higher divergence across species. However, the nuclear tree did not retrieve these as a separate clade. This may be due to lack of variation in the nuclear marker and/or a recent speciation event. Markers which show higher resolution such as microsatellites and AFLPs (Amplified Fragment Length Polymorphism) will be useful in further clarifying this issue. The two distinct groups within PS1 were indeed interesting, however, Rodrigo’s p value for COI was not significant suggesting that there are no cryptic species within PS1. Therefore, these clades within PS1 were not considered as distinct species. The morphology-based tree retrieved these putative species as three distinct morphotypes –2, 3 and 6, falling in different parts of the tree – thus, demonstrating considerable morphological divergence between the putative species. Furthermore, they also exhibit significant differentiation in the environmental axis. The entire group was largely restricted to the southern parts of WG, specifically south of Palaghat Gap (PG) (Fig. 1 and 5), except for a few individuals of PS1 which were from north of PG (Nilgiri Hill range and Kudremukh hill range). In this group, traditional taxonomy recovered two species, *D. coonoorensis* (PS1) and *D. indicus* (PS3). Distinctiveness of PS2 was also established in this study, thus, PS1, PS2 and PS3 are considered three distinct species. Putative species 2 will be henceforth referred to as *D. sp2*.

#### (2) Clade B; PS5 and PS6

Within clade B, PS5 and PS6 were distinct in both mitochondrial and nuclear DNA phylogenies, and the coalescent analysis also recovered them as distinct species with low divergence within and high across the species. Nevertheless, these two PS are not differentiated on morphological and ecological axis. Therefore, morphological and ecological data did not support the splitting of clade B into two species. Interestingly in the morphological analysis, members of clade B grouped with *D. coonoorensis* (PS1) but this clade occupied a very distinct phylogenetic position in the molecular tree. Moreover, members of Clade B (PS5 and PS6) had restricted distribution in the central parts of WG whereas Clade A (which includes PS1) is restricted to southern parts of WG showing complete non-overlap in their distributions. Thus, members of clade B are ecologically as well as genetically distinct from members of the sister Clade (A) and therefore warrant species status (henceforth referred to as *D. spB*). However within Clade B, PS5 and PS6 were not retrieved as distinct species in two out of the three datasets; they have not been considered as putative distinctive species.

#### (3) Clade C; PS7 and PS8

Within Clade C, PS7 and PS8 were divergent on all the three axes, ecology, molecular and morphology. Coalescent analysis also supported their distinctness. PS8 (which according to traditional taxonomy was *D. barnabasi*) was distributed mostly in northern parts of WG whereas PS7 was distributed in the central and southern WG. Thus, it is clear that PS7 represents a new, as of yet unnamed species, referred to in the further descriptions as *D. sp7*.

#### (4) Clade D; PS9

Clade D is represented by a single species PS9, both mitochondrial and nuclear datasets supported its monophyly and the coalescent analysis also recovered it as a distinct species. Morphologically this species was very similar to PS2 (Fig. 4). The predicted distribution of this species was also similar to its current geographic spread but with additional areas. On the PCA plot, where all species were analyzed simultaneously it formed a cluster with members of clade A comprising PS1, PS2 and PS3. This species was distributed in central and southern WG showing sympatry with PS2 and PS3. Traditional taxonomic examination was not useful in assigning this taxonomic unit to any of the previously described species. PS9 shared both ecological and morphological attributes with PS2; nevertheless, it was phylogenetically distinct from members of clade A in that it was sister to all other species (Fig. 2 and 3). Thus, PS9 has been considered as a distinct species (*D. sp9*).

In this paper, *a priori* identification of putative species based on two genes, followed by assessing its status by other independent datasets, such as morphology and environmental data has been successfully illustrated. A new phylogenetic hypothesis, for the *Digitipes* species complex of the WG, with five potentially new species (*D. sp2*, *D. sp4*, *D. spB*, *D. sp7* and *D. sp9*) has been put forward in the current study (Table 2). The actual diversity in this group appears to be twice that of the diversity based on traditional taxonomy. Among these new species at least three were morphologically cryptic species as these species could not be distinguished from either previously described species (*D. spB*) or other new species (*D. sp2 and 9)*. The presences of three morphologically cryptic species – *D. sp2, D. spB* and *D. sp9*– emphasize the need for a multi-disciplinary approach in detecting “hidden” diversity in taxonomically problematic groups. Detection of such morphological cryptic species through a multidisciplinary approach has been reported in varied taxa like bamboo [17], millipedes [39], spiders [9], birds [16], snails [40], ants [41] and lizards [42]. Discovery of cryptic diversity and coarse scale sampling for *Digitipes* in the WG hinted that there is also possibility of unsampled species of *Digitipes* from the WG. Clearly, to attain complete understanding of species diversity in biodiversity rich areas of the tropics, such as in the WG, it is imperative to undertake a multidisciplinary and integrative approach on a range of taxonomic groups. Given the results and the lack of such integrative studies on WG taxa, the probability of species richness in poorly studied groups such as invertebrates and small vertebrates in the WG is being vastly underestimated. In addition, these species pairs show variable divergence along all three axes, highlighting the need to look at many secondary properties of species to understand the process of species formation and speciation.

**Table 2:** Summary of each putative species and support from molecular, morphological and ecological data.

		mtDNA tree(COI & 16S)	Nuclear DNA tree	Separation on Env. Axis(PCA)	Morphology tree	Traditional Taxonomy
**Group A**	PS1	√	χ	√	χ#	*D. coonoorensis*
	PS2	√	χ	√	χ+	*D. indicus*
	PS3	√	χ	√	√	*–*
**Group B**	PS5	√	√	χ	χ#	*–*
	PS6	√	√	χ	χ#	*–*
**Group C**	PS7	√	√	√	√	*–*
	PS8	√	√	√	√	*D. barnabasi*
**Group D**	PS9	√	√	NA	χ+	*–*

√: distinct units; χ: not distinct units; NA: not applicable, χ#: Morphoclade1; χ+: Morphoclade 2.

## Materials and Methods

### Taxon Sampling

Currently six species have been described in genus *Digitipes* from the WG. These include *D. barnabasi, D. coonoorensis, D. gravely, D. pruthii, D. chittonii* and *D. indicus* [38]. Since most of the available published records of these species were from wet evergreen forests of the WG [32], [33], [38], sampling was done across the wet forests of WG encompassing the rainfall and altitudinal gradients present within these forests. The specimen collection permits were obtained from Kerala (PR NO- WL12-2936/2008) and Karnataka (D/WL/CR-23/2008–2010) forest departments to sample in the protected areas. Additionally, some of the adjacent dry forest patches were also searched for the presence of *Digitipes* species. From each locality, only 3–4 individuals were collected. Efforts were also made to collect specimens of each described species from their type locality. A total of 97 specimens belonging to the genus *Digitipes* were collected from 42 localities across the WG. The geographic coordinates of each specimen were also recorded for the purpose of niche modelling. For detailed information on the distribution of sampled specimen see Figure 1 and Table S1.

### Morphological Character Data and Analysis

*Digitipes* species were first identified using the key provided by Jangi and Dass (1984). Furthermore, each specimen was typed for 11 binary morphological characters, also taken from Jangi and Das (1984). These characters included 1) presence (1) or absence (0) of tarsal spur on 20th leg; 2) length of coxopleural process (CP), long-1 and short-0; 3) CP reaches dorsal margin or not, yes-1 and no-0; 4) number of spines on tip of CP (presence of 4 spines-1 and >4 -0 ); 5) presence or absence of lateral spine on CP; 6) number of lateral spines on CP, one-1 or zero-0; 7) to length of distomedial prefemoral process of ultimate leg, long-1 or short-0; 8) presence or absence of spines on (2–20^th^) legs; 9) number of antennal segments 17 (coded as 1) or 19 (coded as 0); 10) number of glabrous segment of antennae, two (coded as 1) or three (coded as 0) and 11) nature of cephalic plate, punctuate-1 and not punctuate-0. This data matrix (Table S3) was used to build a Neighbor-Joining (NJ) tree in PAUP 4.0b2, [43] with “total” distance measure and mid-point rooting. NJ bootstrapping was also carried out to evaluate the support for individual clusters.

### DNA Extraction, Amplification and Sequencing

Genomic DNA extraction was carried out using ethanol preserved tissue samples. In most cases leg tissue was used, but in a few cases segment tissue was also used. The extraction was carried out using standard phenol chloroform method [44]. Partial sequences of two mitochondrial DNA markers –16S rDNA (∼450 bp) and COI (∼600 bp) were generated for 80 individuals using published primers [45] and protocols reported in Joshi and Karanth (2011). The nuclear 28S rDNA (∼350 bp, D3 expansion region) gene was sequenced for a subset of individuals (69 individuals) representing each putative species in mtDNA tree (Table S1).

### Molecular Phylogenetic Analysis

The chromatograms were visualized and edited manually using the program Chromas lite 2.01 (http://www.technelysium.com.au/chromas_lite.html) and then aligned in ClustalW with default settings [46]. Both mtDNA genes, COI and 16S rDNA were aligned separately. They were then concatenated to derive the combined mtDNA dataset. To check for homogeneity in the mtDNA dataset, an Incongruence Length Difference (ILD) test was carried out in WINCLADA [47], [48]. The nuclear 28S rDNA dataset had relatively few parsimony informative sites (14/316), therefore these sequences were aligned and analyzed separately. The program Modeltest [49] was used on these nuclear and mitochondrial datasets for model selection. Both mitochondrial and nuclear datasets were analyzed independently using three different tree building methods, Maximum parsimony (MP), Minimum evolution (ME) and Bayesian approach. Members of genus *Rhysida*, a sister group of the genus *Digitipes* [36] were used as out-group for rooting these trees. Parsimony and Minimum Evolution analyses were carried out in PAUP 4.0b2 through heuristic searches with TBR branch swapping, 10 random-addition replicates, and a random starting tree [43]. For ME analysis, distance was set to GTR based on the Mr. Modeltest output. Parsimony bootstrap supports for the branch nodes were determined through bootstrapping with 1000 replicates and 10 random-addition option. PAUP 4.0b2 settings were the same for both, mitochondrial and nuclear genes. Bayesian analyses were performed in the program MrBayes 2 [50]. The GTR+I substitution model (as chosen by Modeltest) was chosen for 28S and GTR+I+Γ was for both the mitochondrial genes. For mtDNA analysis dataset was portioned according to the genes namely 16S and COI. The program was run for four million generations wherein sampling was done every 100 generations. The standard deviation of split frequencies was used to decide the chain length and the burn-in. For all analyses gaps were treated as missing data.

As mentioned earlier, monophyly of taxa is taken as a proxy for taxonomic distinctiveness [24], but monophyly could arise by chance alone [21], [22]. One can potentially eliminate this scenario by using multiple unlinked markers, wherein nuclear markers also retrieve lineages or units retrieved by mitochondrial marker, thereby confirming taxonomic distinctiveness of these units. However, nuclear markers are often non-informative for closely related and recently diverged species. Two different methods were implemented in this study, on the mitochondrial dataset to test for species monophyly. First, the probability of monophyly for each monophyletic group was evaluated using Rosenberg’s p and Rodrigo’s p [22]. It was done to determine if the observed monophyly was true and not a product of random branching. Secondly, to determine if the observed intra-specific divergence could have arisen solely due to the stochastic process of gene coalescence, another recently developed statistical test was implemented. In this method, a ratio (M) of coalescent intervals, between the tip and species node to between the species node and the common ancestor, is first determined. The probability of obtaining the observed ratio under a standard coalescent model is then calculated. This test assumes that the data follows two principles, a Wright-Fisher population model and strict molecular clock. Recently, ‘species delimitation’ plug-in has been made available to conduct these tests on the tree topology based on single locus data in the program GeneiousPro [51]. A coalescent test for species delimitation recommends use of a one-locus dataset at a time. Therefore, the number of putative species was determined using the two approaches in the ‘species delimitation’ of GeneiousPro on the mitochondrial COI and 16S rDNA dataset respectively.

### Environmental Data and Analysis

For each putative species, identified using the coalescent approach, ecological niche models were developed. A total of 19 bioclimatic layers were obtained from Worldclim Global climate dataset [52] and then extracted only for WG at 1×1 km resolution. These layers and geographic co-ordinates of species were used to predict the distribution using maximum entropy method [53]. This analysis was carried out in MaxEnt version 2.0 with default settings [54]. Model validation was done through Jack-knifing (5 replicates) and randomly splitting dataset (train and test 75∶25) species that had more than 10 localities. This method has been proved to be useful in case of sample sizes as low as 5 [53]. The predicted distribution was visualized on the geographic map using ArcView GIS 3.3. MaxEnt output ranges from 0–100%. Therefore, to make it binary prediction, the lowest presence threshold method was used [55]. In this method, the area with lowest probability value where species presence is observed gets chosen and then used as cut off to make binary predictions. This is a more conservative approach. These predicted distribution maps were created individually for each putative species.

To assess the overlap in the predicted distributions of a species pair, the ratio of overlap between sister species to the smaller species’ range was used. In addition to the geographic overlap, the environmental envelope and its overlap for each species pair was also assessed. To this end, 19 bioclimatic variables have been extracted using locality data of each species and these variables were compiled in ArcView GIS 3.3. These bioclimatic variables were normalized by log_10_ transformation for further statistical analysis. Environmental envelope for each species pair was evaluated by performing Principal Component Analysis (PCA) on the transformed environmental data. To statistically evaluate the overlap in the environmental envelope, multivariate analysis of variance (MANOVA) was used, where species were treated as fixed units with PCA axis scores as the dependent variables. The analysis was done in R 2.1.

## Supporting Information

Table S1 List of specimens along with location and sequence details. Abbreviations: NP – National Park, WLS – Wildlife Sanctuary, RF – Reserve Forests Location in bold indicate the type locality/very close to type locality sampled for the species.
(DOCX)

Table S2 Summarized coalescent analysis results for 16S rDNA and COI.
(DOCX)

Table S3 Morphology character matrix for the *Digitipes* specimens. Abbreviations: 1) presence or absence of tarsal spur on 20th leg; 2) length of coxopleural process (CP), 3) CP reaches dorsal margin or not; 4) number of spines on tip of CP; 5) presence or absence of lateral spine on CP; 6) number of lateral spines on CP; 7) length of distomedial prefemoral process of ultimate leg; 8) presence or absence of spines on (2–20^th^ ) legs; 9) number of antennal segments 17 or 19; 10) number of glabrous segment of antennae, two or three and 11) nature of cephalic plate.
(DOCX)
