# Supplementary material for: Coalescent Method in Conjunction with Niche Modeling Reveals Cryptic Diversity among Centipedes in the Western Ghats of South India
Source: PLoS One. 2012 Aug 2;7(8):e42225. doi: 10.1371/journal.pone.0042225 (PMC3410927; doi:10.1371/journal.pone.0042225)
Supplement: Table S1 — List of specimens along with location and sequence details. Abbreviations: NP – National Park, WLS – Wildlife Sanctuary, RF – Reserve Forests Location in bold indicate the type locality/very close to type locality sampled for the species. (DOCX) [file pone.0042225.s001.docx]

| Species | Specimen number | Lat | Long | COI | 16S | 28S |
| --- | --- | --- | --- | --- | --- | --- |
| *D. coonoorensis* | **CES07125** | **11.20217** | **76.43915** | **JN004032** | **JN003921** | **JN003976** |
|  | **CES07132** | **11.2037** | **76.43872** | **JN004035** | **JN003924** | **JN003979** |
|  | **CES07134** | **11.20894** | **76.44114** | **JN004037** | **JN003926** | **JN003981** |
|  | CES08921 | 13.12976 | 75.27582 | + | + | + |
|  | CES08960 | 10.17562 | 77.10619 | + | + | + |
|  | CES08980 | 10.18347 | 77.08972 | + | + | + |
|  | CES08987 | 10.19129 | 77.09736 | + | + | + |
|  | CES08992 | 10.21493 | 77.09435 | + | + | + |
|  | CES08994 | 10.21871 | 77.0894 | + | + | - |
|  | CES091088 | 8.87275 | 77.1634 | + | + | + |
|  | CES091334 | 10.98198 | 76.64134 | + | + | + |
| *D. sp2* |  |  |  |  |  |  |
|  | CES07157 | 8.67808 | 77.15989 | JN004039 | JN003928 | JN003983 |
|  | CES07158 | 8.67917 | 77.16057 | + | + | + |
|  | CES07160 | 8.67989 | 77.15891 | JN004040 | JN003929 | JN003984 |
|  | CES07161 | 8.67989 | 77.15891 | JN004041 | JN003930 | JN003985 |
|  | CES07162 | 8.68164 | 77.16292 | JN004042 | JN003931 | JN003986 |
|  | CES07166 | 8.66625 | 77.17135 | JN004043 | JN003932 | JN003987 |
|  | CES07169 | 8.66444 | 77.17878 | JN004044 | JN003933 | JN003988 |
|  | CES07171 | 8.65853 | 77.17789 | JN004045 | JN003934 | JN003989 |
|  | CES07173 | 8.66258 | 77.17117 | JN004046 | JN003935 | JN003990 |
|  | CES07174 | 8.66436 | 77.16897 | JN004047 | JN003936 | JN003991 |
|  | CES07196 | 8.75552 | 77.11374 | + | + | + |
|  | CES07197 | 8.75615 | 77.11544 | + | + | + |
|  | CES07198 | 8.75708 | 77.11667 | + | + | + |
|  | CES091086 | 8.87501 | 77.15968 | + | + | + |
|  | CES091087 | 8.87313 | 77.16277 | + | + | + |
|  | CES091089 | 8.87323 | 77.16664 | + | + | - |
|  | CES091090 | 8.87313 | 77.16277 | + | + | - |
|  | CES091091 | 8.87275 | 77.1634 | + | + | - |
|  | CES091096 | 8.86419 | 77.17968 | + | + | - |
|  | CES091304 | 9.1072 | 77.1306 | + | + | - |
|  | CES091305 | 9.1072 | 77.1306 | + | + | - |
|  | CES091324 | 9.07032 | 77.20628 | + | + | - |
|  | CES091325 | 9.07089 | 77.20661 | + | + | + |
|  | CES091326 | 9.07193 | 77.20597 | + | + | - |
| *D. indicus* |  |  |  |  |  |  |
|  | CES07168 | 8.66444 | 77.17878 | + | + | - |
|  | CES07183 | 8.72999 | 77.12369 | + | + | + |
|  | CES08982 | 10.44624 | 76.81662 | + | + | + |
|  | CES08990 | 10.20795 | 77.09597 | + | + | + |
|  | CES08996 | 10.13224 | 76.68311 | + | + | + |
|  | CES08997 | 10.13224 | 76.68311 | + | + | + |
|  | CES08998 | 10.13379 | 76.68188 | + | + | + |
|  | CES091005 | 10.57543 | 76.38538 | + | + | - |
|  | CES091006 | 10.57543 | 76.38538 | + | + | + |
|  | CES091008 | 10.52727 | 76.35003 | + | + | + |
|  | **CES091047** | **10.3945** | **76.66923** | **+** | **+** | **+** |
|  | **CES091049** | **10.37976** | **76.65624** | **+** | **+** | **+** |
|  | CES091057 | 10.30995 | 76.72245 | + | + | + |
|  | CES091062 | 10.30152 | 76.60533 | + | + | + |
|  | CES091310 | 9.44292 | 76.9835 | + | + | - |
|  | CES091315 | 9.44304 | 76.97916 | + | + | - |
|  | CES091316 | 9.4406 | 76.98152 | + | + | - |
|  | CES091318 | 9.4406 | 76.98152 | + | + | - |
|  | CES091319 | 9.44241 | 76.98309 | + | + | - |
|  | CES091322 | 9.42278 | 76.98937 | + | + | - |
|  |  |  |  |  |  |  |
| *D. spB* |  |  |  |  |  |  |
|  | CES07219 | 15.27252 | 74.9603 | JN004048 | JN003937 | JN003992 |
|  | CES07223 | 14.98779 | 74.37476 | JN004049 | JN003938 | JN003993 |
|  | CES07226 | 14.98968 | 74.37769 | JN004050 | JN003939 | JN003994 |
|  | CES07230 | 14.98897 | 74.37122 | JN004051 | JN003940 | JN003995 |
|  | CES07233 | 14.51634 | 74.54063 | JN004052 | JN003941 | JN003996 |
|  | CES07288 | 12.38891 | 75.49002 | + | + | + |
|  | CES08907 | 13.20181 | 75.19191 | + | + | + |
|  | CES08912 | 13.20052 | 75.19289 | + | + | - |
|  | CES08915 | 13.20181 | 75.19191 | + | + | + |
|  | CES08922 | 13.13392 | 75.2782 | + | + | + |
|  | CES08930 | 17.91192 | 73.63675 | + | + | - |
|  | CES091020 | 12.72381 | 75.70969 | + | + | + |
| *D. sp7* |  |  |  |  |  |  |
|  | CES07138 | 11.20894 | 76.44114 | + | + | + |
|  | CES08911 | 13.20076 | 75.19249 | + | + | + |
|  | CES091017 | 12.4055 | 75.51992 | + | + | + |
|  | CES091033 | 9.5755 | 77.33617 | + | + | + |
|  | CES091039 | 9.58402 | 77.34902 | + | + | + |
|  | CES091073 | 8.86194 | 77.17951 | + | + | - |
|  | CES091341 | 9.07202 | 77.19042 | + | + | - |
| *D. barnabasi* |  |  |  |  |  |  |
|  | CES07210 | 18.4189 | 73.9068 | + | + | + |
|  | CES07215 | 15.3698 | 74.3063 | + | + | + |
|  | CES07237 | 14.52567 | 74.60246 | + | + | + |
|  | CES07244 | 15.3868 | 74.3192 | + | + | + |
|  | CES07284 | 18.41448 | 73.90362 | + | + | + |
|  | CES08932 | 17.91572 | 73.6363 | + | + | - |
|  | CES08953 | 15.95936 | 73.99971 | + | + | - |
|  | CES08957 | 15.96171 | 74.00206 | + | + | + |
|  | CES091016 | 12.4055 | 75.51992 | + | + | + |
|  | **CES091345** | **19.06325** | **73.53964** | **+** | **+** | **+** |
|  | **CES091348** | **19.07772** | **73.55047** | **+** | **+** | **+** |
|  | **CES091353** | **19.13194** | **73.57083** | **+** | **+** | **+** |
|  | **CES091355** | **19.13214** | **73.57061** | **+** | **+** | **+** |
| *D. sp9* |  |  |  |  |  |  |
|  | CES091013 | 12.66 | 75.6836 | + | + | + |
|  | CES091021 | 12.73057 | 75.66403 | + | + | - |
|  | CES091048 | 10.38819 | 76.66116 | + | + | + |
|  | CES091327 | 10.96864 | 76.66691 | + | + | + |
|  | CES091330 | 10.97176 | 76.65796 | + | + | + |
|  | CES091331 | 10.97165 | 76.65794 | + | + | - |
|  | CES091342 | 9.07171 | 77.19156 | + | + | + |
