# Supplementary material for: Coalescent Method in Conjunction with Niche Modeling Reveals Cryptic Diversity among Centipedes in the Western Ghats of South India
Source: PLoS One. 2012 Aug 2;7(8):e42225. doi: 10.1371/journal.pone.0042225 (PMC3410927; doi:10.1371/journal.pone.0042225)
Supplement: Table S2 — Summarized coalescent analysis results for 16S rDNA and COI. (DOCX) [file pone.0042225.s002.docx]

| **COI** | | | | | | | |
| --- | --- | --- | --- | --- | --- | --- | --- |
| Species | Closest Species | Monophyletic? | Intra Dist | Inter Dist - Closest | Intra/Inter | P(Randomly Distinct) | Rosenberg's P(AB) |
| PS1 | PS4 | yes | 0.074 | 0.158 | 0.47 | 1 | 3.90E-13 |
| PS2 | PS1 | yes | 0.033 | 0.18 | 0.18 | 0.05 | 7.80E-14 |
| PS3 | PS1 | yes | 0.076 | 0.163 | 0.47 | 1 | 7.80E-14 |
| PS5 | PS4 | yes | 0.055 | 0.148 | 0.37 | 1 | 0.02 |
| PS4 | PS5 | yes | 0.004 | 0.148 | 0.03 | < 0.05 | 0.02 |
| PS6 | PS4 | yes | 0.112 | 0.149 | 0.75 | 0.98 | 4.40E-05 |
| PS9 | PS1 | yes | 0.086 | 0.194 | 0.45 | 1 | 1.20E-10 |
| PS7 | PS8 | yes | 0.152 | 0.182 | 0.83 | 0.83 | 4.00E-06 |
| PS8 | PS7 | yes | 0.108 | 0.182 | 0.59 | 1 | 4.00E-06 |
| **16S** | | | | | | | |
| Species | Closest Species | Monophyletic? | Intra Dist | Inter Dist - Closest | Intra/Inter | P(Randomly Distinct) | Rosenberg's P(AB) |
| PS1 | PS4 | yes | 5.444 | 10.55 | 0.52 | 0.05 | 7.10E-09 |
| PS3 | PS1 | yes | 7.211 | 11.75 | 0.61 | 0.05 | 7.10E-09 |
| PS2 | PS4 | yes | 8.373 | 11.29 | 0.74 | 1 | 4.90E-17 |
| PS5 | PS6 | yes | 3.8 | 7.9 | 0.48 | 1 | 1.20E-04 |
| PS6 | PS5 | yes | 4.786 | 7.9 | 0.61 | 1 | 1.20E-04 |
| PS7 | PS4 | no | 5.048 | 8 | 0.63 | NA | NA |
| PS8 | PS7 | yes | 6.051 | 8.648 | 0.7 | 1 | 1.30E-05 |
| PS4 | PS9 | yes | 2 | 7.286 | 0.27 | 0.9 | 6.00E-06 |
| PS9 | PS4 | yes | 4.571 | 7.286 | 0.63 | 0.97 | 1.90E-12 |
